# Tracking Vaccination Teams During Polio Campaigns in Northern Nigeria by Use of Geographic Information System Technology: 2013–2015

**DOI:** 10.1093/infdis/jiv493

**Published:** 2016-04-02

**Authors:** Kebba Touray, Pascal Mkanda, Sisay G. Tegegn, Peter Nsubuga, Tesfaye B. Erbeto, Richard Banda, Andrew Etsano, Faisal Shuaib, Rui G. Vaz

**Affiliations:** 1 World Health Organization, Country Representative Office, Abuja, Nigeria; 2 World Health Organization, Regional Office for Africa, Brazzaville, Congo; 3 Global Public Health Solutions, Atlanta, Georgia

**Keywords:** tracking vaccination teams, dashboard, team performance, geographic coverage of settlements

## Abstract

***Introduction.*** Nigeria is among the 3 countries in which polio remains endemic. The country made significant efforts to reduce polio transmission but remains challenged by poor-quality campaigns and poor team performance in some areas. This article demonstrates the application of geographic information system technology to track vaccination teams to monitor settlement coverage, reduce the number of missed settlements, and improve team performance.

***Methods.*** In each local government area where tracking was conducted, global positioning system–enabled Android phones were given to each team on a daily basis and were used to record team tracks. These tracks were uploaded to a dashboard to show the level of coverage and identify areas missed by the teams.

***Results.*** From 2012 to June 2015, tracking covered 119 immunization days. A total of 1149 tracking activities were conducted. Of these, 681 (59%) were implemented in Kano state. There was an improvement in the geographic coverage of settlements and an overall reduction in the number of missed settlements.

***Conclusions.*** The tracking of vaccination teams provided significant feedback during polio campaigns and enabled supervisors to evaluate performance of vaccination teams. The reports supported other polio program activities, such as review of microplans and the deployment of other interventions, for increasing population immunity in northern Nigeria.

The 41st World Health Assembly's resolution in 1988 for the worldwide eradication of polio marked the launch of the Global Polio Eradication Initiative (GPEI), spearheaded by national governments, the World Health Organization (WHO), Rotary International, the Centers for Disease Control and Prevention, and the United Nations Children's Fund and supported by key partners [1]. The GPEI was based on 4 strategies: high coverage with oral polio vaccine in routine immunization, surveillance for acute flaccid paralysis, supplemental immunization activities (SIAs), and mop-up immunizations. Nigeria made significant efforts but remained endemic for wild poliovirus (WPV) as a result of several factors but mainly because of poor quality and coverage of the SIAs. Another hindrance was the inability to identify areas that were missed by timely intervention with vaccination to provide the required herd immunity to break the polio transmission.

The government of Nigeria, through the national and state emergency polio operations centers (EOCs), in collaboration with the WHO and partners, deployed geographic information system (GIS) technology to track vaccination teams during polio campaigns. These innovations are now increasingly used by public health professionals to visualize and explore disease patterns [2–5]. Similar tools were also used in India to monitor population migration through railroads, and Google Earth was used to track the poliovirus down the Congo River [6, 7]. These studies have demonstrated that GIS is an essential tool for making informed decisions on health, social, and environmental issues. In Nigeria, global positioning system (GPS) and GIS solutions were deployed in 2010 and piloted in the tracking of vaccination teams and for supporting the microplanning process for SIAs [8, 9]. Since 2010, GIS has been part of an intense package of interventions in Nigeria and has contributed to the successes so far recorded. The quality of the immunization campaigns has significantly improved, and the country has recorded a significant decline in the number of confirmed cases of WPV infection between 2012 and 2015, from 122 cases to 0. This article describes the innovative use of GIS in tracking vaccination teams during SIAs.

## METHODS

### Study Area and Population

Vaccination tracking and mapping was conducted in 10 states at high risk for polio transmission (ie, states at high risk for WPV transmission owing to history of infection and quality of campaigns therein) in northern Nigeria (ie, Bauchi, Borno, Jigawa, Kaduna, Kano, Kebbi, Katsina, Sokoto, Yobe, and Zamfara). The 2015 projected population in these states is 62 172 215 people.

### Data Collection and Mapping

Beginning in 2012, extensive mapping of these 10 states was conducted to generate ward-level (where ward is the lowest administrative level) GIS maps with information on settlements and other points of interest, such as health facilities, schools, and markets. Data collection was conducted through a combination of fieldwork and feature extraction, using satellite imagery. Personnel at both local government area (LGA) and ward levels with extensive local knowledge were trained on field data collection. The maps generated provided the background data for the use of GIS maps for microplanning and also facilitated the tracking of vaccination team members during SIAs.

### Tracking Vaccination Teams

To track vaccination teams during SIAs and visualize settlement coverage, a vaccination tracking system (VTS) dashboard was developed [10]. In the VTS dashboard, settlements were categorized into urban areas (ie, large settlements or cities with >100 residences), small-settlement areas (ie, rural villages with 20–100 residences), and hamlet areas (ie, clusters of hamlets, with each hamlet containing <20 residences).

### Vaccination Tracking Process

Owing to the technical capacity requirements of tracking vaccination teams, tracking was limited to a few LGAs each round. In 2013, 40 LGAs were tracked each round, and this increased to 60 in 2014 and 80 by 2015. The selection of LGAs for tracking was based on the priorities of the polio program. Priority for tracking vaccination teams was given mainly to LGAs that were in the high-risk category and also to LGAs with recent circulating vaccine-derived poliovirus or WPV transmission. The national EOC, in collaboration with the state EOCs, approved the list of LGAs to track at each round; this approved list was then shared with the GIS team for setting up the tracking phones and the VTS required for uploading and displaying the tracking results.

During each day of the polio campaign, GPS-enabled Android phones were given to a ward focal person at LGA headquarters, who distributed the phones to vaccinators at the ward takeoff point. At the end of each day, the tracks, which were made up of data gathered automatically from the movement of vaccination teams while their GPS was turned on and provided a record of where and when they traveled, were uploaded to the local laptop at the LGA and then to the EOC's dashboard via a wireless router acting as a mobile wireless hot spot. Feedback on results of the daily coverage displayed on the dashboard was provided to ward focal persons and the LGA team at daily review meetings. Furthermore, a missed or poorly covered settlement report was generated at the end of day 4 of the SIA and shared with the field team. The partially covered and missed settlement reports showed all settlements in which the cumulative percentage of areas visited was below a certain threshold. A settlement was considered partially covered if <70%, 50%, and 100% of the urban, hamlet, and small-settlement areas, respectively, were covered with tracks. On the other hand, urban, hamlet, and small-settlement areas with coverage of <50%, <40%, and <1%, respectively, were classified as missed. At the end of day 5 of the SIA, the missed settlement report was validated at 3 different levels to ensure that the final postcampaign list included only settlements not visited by vaccination teams. An automated vaccination tracking report after the campaign was also generated. The identified missed areas were then visited for a focused vaccination.

### Estimation of Geographic Coverage

To estimate the geographic coverage for all the settlement types, GPS positions were compared to positions at the following smallest denominators: urban areas were divided in 50 × 50-m grids, small settlements were identified as GPS points with a 75-m buffer around them, hamlets were identified as GPS points with a 50-m buffer around them, and hamlet areas were delineated on the basis of clusters of hamlets within 200 m from one another.

The geographic coverage data were analyzed to show 2 key indicators: (1) the cumulative percentage visited (for the various settlement types), defined as the percentage of smallest denominators (ie, grid squares for urban areas or buffered points for small settlements and hamlets) that have intersected at least once with a team's GPS tracks; and (2) the total percentage visited, calculated as the average cumulative percentage visited across all settlement types.

GPS positions were collected every 2 minutes, but only GPS tracks that satisfied the following rules were considered valid: for urban and small-settlement areas, tracks were made within the campaign days, were made between 5 am and 6 pm, and had a speed at the capture time of <1 m/second; and for hamlet areas, tracks were made within the campaign days and between 5 am and 6 pm.

## RESULTS

**Figure 1.**
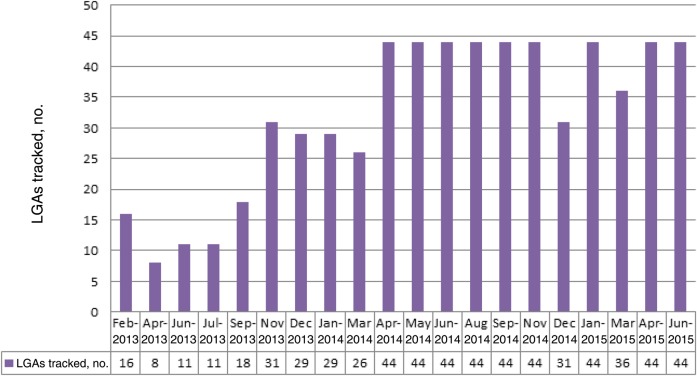
Local government areas (LGAs) tracked, by campaign month, Kano state during February 2013 through June 2015 in northern Nigerian states mapped by global information system technology.

Tracking activities, which started in October 2012 as a pilot, were conducted in 8 of 10 GIS-mapped states in northern Nigeria (Table 1). During this pilot phase, from October to December 2012, 25 LGAs in Jigawa, Kano, Katsina, Sokoto and Zamfara states were tracked. Between October 2012 and June 2015, tracking of vaccination team members covered 119 immunization plus days (IPDs) between these states including mop-ups, which were conducted mainly in Kano state during outbreak response activities. There were 1149 tracking activities conducted during October 2012–June 2015, of which 681 (59%) were conducted in Kano. Figure 1 shows the number of LGAs tracked during each SIA round in Kano state, the epicenter of polio transmission, during the 20 rounds of polio campaigns from April 2013 to June 2015. From April 2014 onward, all LGAs in the state were tracked each round, except during December 2014 and March 2015, when inactivated polio vaccine was administered in health camps in some LGAs, which therefore did not participate in the house-to-house campaign.

**Table 1.** Number of Local Government Authorities (LGAs) Tracked During October 2012–June 2015 in Northern Nigerian States Mapped by Global Information System Technology

State	LGAs	IPDs	Oct–Dec 2012	Jan–Dec 2013	Jan–Dec 2014	Jan–Jun 2015	Total
Bauchi	20	9	0	0	10	11	21
Borno	27	1	0	2	0	0	2
Jigawa	27	12	8	6	19	30	63
Kano	44	28	8	130	375	168	681
Katsina	34	21	3	42	52	33	130
Kebbi	21	7	0	5	40	17	62
Sokoto	23	23	4	41	54	27	126
Zamfara	14	18	2	18	10	34	64
Total	210	119	25	244	560	320	1149

Abbreviations: IPD, immunization plus day; LGA, local government area.

**Figure 2.**
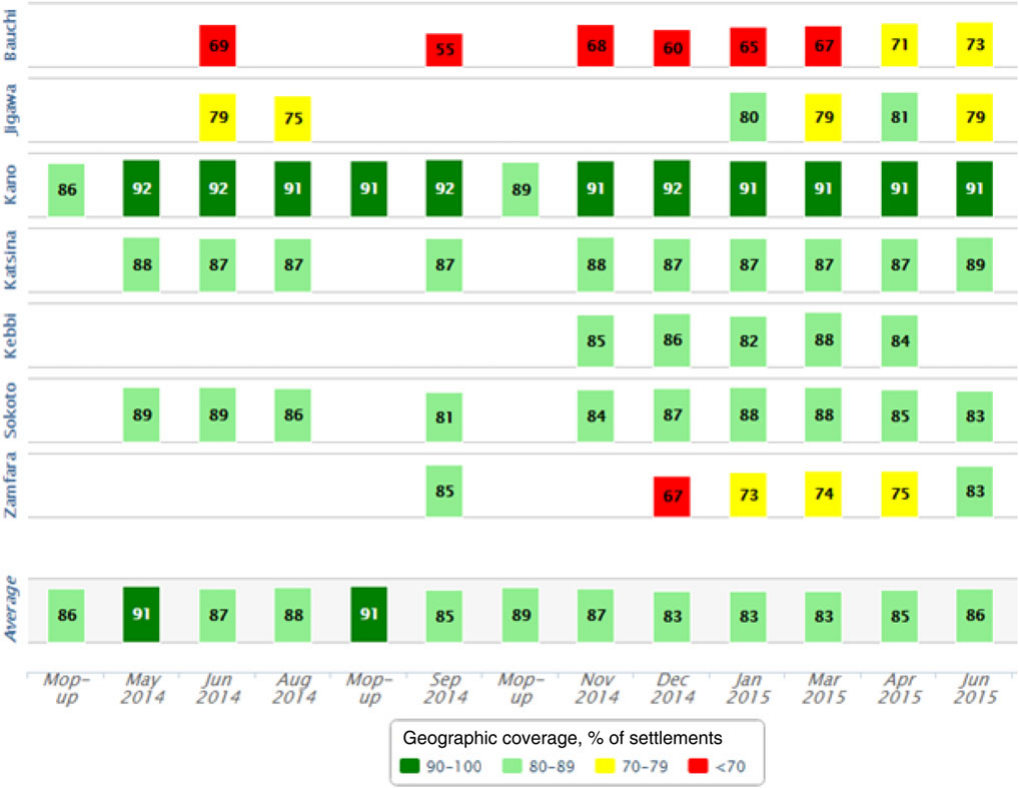
Vaccination tracking geographic coverage, by Nigerian state and month, during May 2014–June 2015. Figure from Vaccination Tracking System.

**Figure 3.**
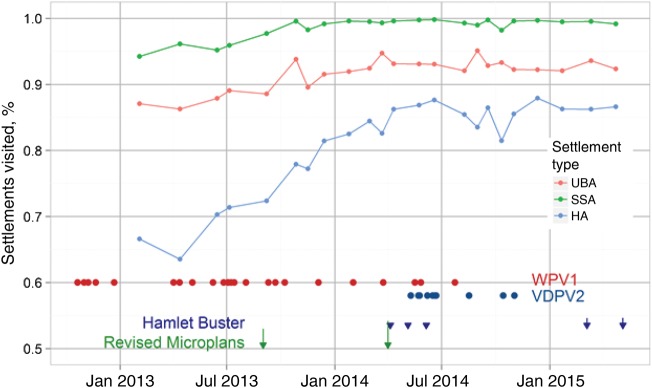
Proportion of settlements in Kano state visited over time, by settlement type. “Hamlet Buster” was an activity that was conducted to locate and name hamlets using tablets uploaded with ward maps. Abbreviations: HA, hamlet area; SSA, small-settlement area; UBA, urban area; VDPV2, vaccine-derived poliovirus type 2; WPV1, wild poliovirus type 1. Figure from A. Upfill-Brown, Institute for Disease Modeling.

Figure 2 shows the geographic coverage of vaccination tracking by state and month, during May 2014–June 2015. During this period, tracking was done in 7 of 10 states and included 3 mop-up campaigns conducted in Kano state. This coverage report, which was automated in the VTS, categorized the geographic coverage into 4 coverage bands ranging from ≥90% to <70%. All states improved coverage over time, with the coverage for Kano state—the most tracked state—also increasing (Figure 3).

From the missed settlements results presented in Table 2 for 2014 and 2015, there was a reduction in the number of settlements missed during polio campaigns in Kano, Kebbi, and Sokoto states. However, an increase in the number of missed settlements was recorded in Bauchi and Zamfara states during the same period. Jigawa and Katsina states also had a slight increase in the number missed. Also, in Table 2, the number of chronically missed settlements (ie, settlements that had been consistently missed in the last 3 polio campaigns) decreased significantly for all states except Zamfara, which had a slight reduction between 2014 and 2015. In Kano state, the number of chronically missed settlements decreased from 1298 in 2014 to 165 in 2015, showing a huge drop in missed settlements.

**Table 2.** Number of Missed and Chronically Missed Settlements, by State: 2014 and 2015, Northern Nigeria

State	Missed		Chronically Missed	
	2014	2015	2014	2015
Bauchi	1567	2358	1884	219
Jigawa	187	260	750	166
Kano	411	152	1298	165
Katsina	211	230	441	80
Kebbi	351	157	742	295
Sokoto	176	52	361	34
Zamfara	1239	3799	357	298

**Figure 4.**
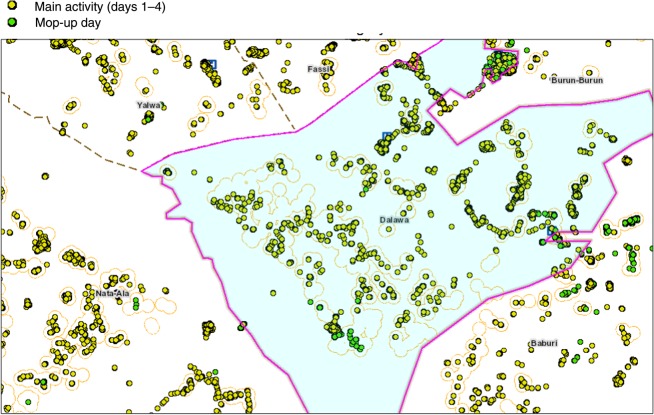
Areas visited by vaccination teams during polio campaigns in northern Nigeria, highlighting main vaccination activities (days 1–4 of campaigns) and mop-up activities (day 5; final campaign day). Figure from Vaccination Tracking System.

Figure 4 shows the settlements covered by vaccination teams during polio campaigns in northern Nigeria, highlighting main vaccination activities (days 1–4 of campaigns) and mop-up activities (day 5; final campaign day). The map shows how the vaccination tracking information is used to identify missed settlements and then verify coverage in these areas at the end of mop-up vaccinations.

## DISCUSSIONS

We have demonstrated that routine tracking of vaccination teams during polio campaigns resulted in improved geographic coverage particularly in Kano, the most tracked state and the epicenter of polio transmission in northern Nigeria. Tracking of vaccination teams provided significant feedback on settlements reached or completely missed and thereby enabled supervisors to evaluate performance and missed settlements to be revisited in real time in the form of mop-up vaccinations. In addition, monitoring vaccination teams' movement through tracking made vaccinators accountable throughout the campaigns. The overall goal of tracking is to reach every child and settlement regardless of size and distance.

GIS tracking of vaccination teams has become an important monitoring tool for improving immunization coverage for the Nigeria polio program. This is because the effectiveness of immunization is best when a larger proportion of the population is protected against the disease by vaccination, through herd immunity. As long as there are significant pockets of vulnerable populations, such as chronically missed settlements, poliovirus transmission will continue to occur. Our experience shows that, between 2013 and 2015, there was a reduction in missed and chronically missed settlements for many states. These significant changes could have contributed in improving the population immunity, thereby breaking the chain of polio transmission. However, the high numbers of missed settlements from the LGAs tracked in Zamfara and Bauchi states were attributed mainly to compromised security, as some settlements were not accessible to vaccination teams during SIAs, owing to safety concerns. This is an indication that tracking can show areas that are doing well, highlight places in which coverage has been generally low, and provide reasons for such low coverage.

Furthermore, the outputs from vaccination tracking were used to support a number of activities in the tracked states that were relevant for polio eradication. These included the use of smart phones and tablets to locate chronically missed settlements and unnamed hamlet areas, to review microplans and align the list of settlements in the GIS database with the ward-level microplan settlement list, and to deploy other interventions in settlements that were missed and inaccessible owing to security concerns during SIAs as per the tracking results. Because of the visualization capability of the tracking system, supervisors used the coverage maps alongside the various automated reports to determine whether mop-up immunization activities were effective. Overall, we observed that improvements in settlement coverage, especially among hamlet areas, correlated with the disappearance of WPV type 1 from the state and with a significant increase in other polio program activities in Kano state.

While we have shown the importance of GIS tracking of vaccination teams during polio campaigns in northern Nigeria, the use of this innovation has some limitations. First, although the tracking provides important feedback on the settlements reached by the various vaccination teams using the different reports, recording and analyzing geographic coverage does not translate into actual vaccination of children in the settlements reached. Some areas may achieve a high geographic coverage but record a high number of missed children, which could be attributable to noncompliance, children being absent, and teams' attitudes. We validated this by performing spontaneous and random checks on the areas covered and missed. In addition, there is a huge resource capacity requirement to track all vaccination teams, which was an important reason tracking was limited to only 80 LGAs in the north.

Despite these limitations, we observed that the use of GIS tracking, coupled with other innovations, has contributed to the successes so far recorded in Kano and other tracked states. Routine tracking of vaccination teams was effective in ensuring that teams reached the settlements they were supposed to cover and enabling supervisors to monitor the level of coverage. In conclusion, the use of GIS, as demonstrated in this study, is highly valuable in improving population immunity. It is a useful analytical tool for incorporation into health programs, especially in microplanning for polio campaigns, routine immunization, disease surveillance, and response systems. The resource requirements should not prevent sustaining the use of the innovation, since most of the financial resources support the mapping of the 10 states. With these data already available and local capacity in place to handle the various components of the vaccination tracking system, tracking activities can always be conducted and the innovation could extend beyond the polio program to other health interventions. The required additional resources, which are less than the initial cost of building the system, could be available through government funding and not necessarily dependent on external donor funds.

GIS technology, if used effectively alongside other interventions, could contribute immensely to the interruption of polio in Nigeria. We recommend that, to ensure accountability and improve team performance in line with the polio endgame strategies, tracking of teams during polio campaigns should be sustained. The tracking reports should also be routinely used in the microplanning verification exercises, as this will assist in identifying settlements that are captured in the GIS database but not the microplans. A regular report using the time-stamped information should be added to the tracking output reports, to determine the time spent by each team during each campaign day. Such reports, if presented during review meetings and acted upon, may also improve team efficiency during campaigns.
